# A Safe Way to Administer Drugs Through a Nutrition Tube—The Simple Suspension Method

**DOI:** 10.1007/s00455-021-10280-w

**Published:** 2021-03-14

**Authors:** Kenjiro Kunieda, Naomi Kurata, Yuki Yoshimatsu, Tomohisa Ohno, Takashi Shigematsu, Ichiro Fujishima

**Affiliations:** 1 Department of Neurology, Gifu University Graduate School of Medicine, 1-1 Yanagido, Gifu, 501-1194 Japan; 2 Department of Rehabilitation Medicine, Hamamatsu City Rehabilitation Hospital, Hamamatsu, Japan; 3 School of Pharmacy, Showa University, Tokyo, Japan; 4 Department of Respiratory Medicine, Iizuka Hospital, Fukuoka, Japan; 5 Department of Physiology, Hyogo College of Medicine, Nishinomiya, Japan; 6 Department of Dentistry, Hamamatsu City Rehabilitation Hospital, Hamamatsu, Japan

**Keywords:** Deglutition, Deglutition disorders, Clogging, Tablets, Tube feeding, Warm water

## Abstract

The simple suspension method (SSM), developed by Kurata in 1997, is a way to suspend tablets and capsules in warm water for decay and suspension prior to their administration. This method is safe and has various advantages such as the avoidance of tube clogging and the loss of the drug. This study aimed to investigate whether a higher percentage of commonly used drugs could pass through nutrition tubes effectively using SSM, relative to that using the conventional crushing method. A tablet or capsule was inserted into a 20 mL syringe with warm water (at 55 °C). After 10 min, it was shaken in the syringe. The suspension liquid was injected into tubes of the following sizes: 8 Fr, 10 Fr, 12 Fr, 14 Fr, 16 Fr, and 18 Fr. A total of 3686 tablets and 432 capsules that are frequently used in Japan were tested. Using SSM, 3377 (91.6%) tablets and 359 (83.1%) capsules disintegrated within 10 min and passed through the tube without clogging it in the tube passage test. With the conventional crushing method, 2117 tablets (57.4%) and 272 capsules (63.0%) could be crushed. SSM reduced the risk of tube clogging and drug loss with more drugs than that with the conventional crushing method. The number of drugs indicated for administration by SSM is greater than that indicated by the conventional crushing method. Further studies are needed to consider its utility compared to conventional methods for dysphagia patients in clinical settings.

## Introduction

Patients with dysphagia often depend on nasogastric or percutaneous endoscopic gastrostomy (PEG) tubes for their daily nutrition, hydration, and medication. It is common for these patients to have difficulty in swallowing tablets, and clinicians must choose the drug form and administration route according to each patient’s swallowing function. In patients dependent on nasogastric or PEG tubes, drugs are often crushed into a powder and suspended in water to enable its passage through the tubes.

Typically, when drugs are crushed, multiple tablets are crushed together and then mixed and put in one sachet. Although these crushing methods are commonly used in daily clinical settings, there are many problems associated with them. For example, it can clog a tube during administration. It has been reported that nasogastric tubes can get clogged in 6–45% of cases [1–8]. Crushing drugs can also lead to the loss of drugs. Drugs tend to attach to the grinding device or medicine paper during crushing, in addition to being left in cups or syringes while preparing suspensions. Furthermore, the crushing of tablets or the opening of capsules can scatter the drug and cause a higher risk of toxicity if healthcare professionals contact or inhale them. Moreover, crushing drugs is a tedious and time-consuming task, particularly for healthcare professionals such as pharmacists or nurses. When multiple crushed drugs are mixed for administration to patients with dysphagia, it is impossible to selectively remove one drug or replace it with another. Thus, all contents must be discarded if it becomes necessary to change the drugs after dispensing. This results in an increased workload and also an economic loss.

In Japan, in 1997, to overcome these issues associated with drug administration, one of the authors of this study (Kurata) developed the original method to suspend tablets and capsules in warm water instead of crushing them. This method, called the simple suspension method (SSM), is now widely utilized throughout the country. Kurata and Fujishima published the first book on SSM, which is used not only in hospitals but also in nursing and home care facilities in Japan [9]. The SSM has been approved by the Japanese medical insurance system with the medical fee revisions in 2020. There have been reports providing an overview of enteral feeding tubes, drug administration techniques, and methods to minimize tube occlusions [10, 11]. The effectiveness of SSM has been reported for a limited number of drugs [12, 13]. However, there have not been any comprehensive reports on the efficacy of suspension methods for routinely used drugs in clinical settings.

We hypothesized that commonly used drugs could effectively pass through nutrition tubes at a higher percentage when using the SSM than when using the conventional crushing method. Therefore, this study aimed to investigate whether higher percentages of commonly used drugs could pass through nutrition tubes using SSM relative to that using the conventional crushing method.

## Methods

### Time and Water Temperature

When suspended in water, many tablets disintegrated in a short time, often within a few minutes. For SSM, we allocated a suspension time of 10 min to allow enough time for the drug to disintegrate. Drugs that did not disintegrate in 10 min were cracked to enable easy penetration water, excluding specific drugs such as slow-release tablets. In the Japan Pharmacopoeia, capsules are formulated to dissolve within 10 min when shaken in 50 mL of water at 37 ± 2 °C [13]. As it is difficult to maintain the water at 37 °C for 10 min, we decided to determine the lowest temperature of warm water that remained above 37 °C for more than 10 min at room temperature. Cups were filled with 20 mL of warm water at different temperatures to observe the change in temperature. At a room temperature of 24 °C, water at 50 °C was 36 °C after 10 min, which was < 37 °C. Water at 55 °C was around 40 °C after 10 min. Therefore, the water temperature of SSM was set at 55 °C.

### Suspension Test

The plunger of a 20 mL syringe was pulled out, and one tablet or capsule was placed inside before putting the plunger back in place. 20 mL of water at 55 °C was introduced into it, and it was left at room temperature for 5 min, with the cap replaced back onto the tip. The syringe was then rotated back and forth at a 180° angle to stir the formula, and the degree of the drug’s decay and suspension status was observed. If it was inadequate, the syringe was left stationary for another 5 min, and then turned at a 180° angle 15 times to stir it. If the disintegration was still inadequate after 10 min and stirring, the operation was discontinued. Regarding the drugs that were discontinued, interview forms which contained information from drug companies were checked to determine whether crushing or decapsulation was allowed [14, 15]. Interview forms are more detailed explanatory documents compared with the drug package inserts supplied to pharmacists, which the Japan Hospital Pharmacists Association commissioned pharmaceutical companies to prepare and distribute. If coated tablets permitted crushing, the coating was destroyed by cracking the drug over a sheet with a pestle before placing the tablet in the syringe. If decapsulation was permitted, the capsule was opened, and the drug inside was placed in the syringe using the same method. Drugs that did not disintegrate and were not permitted to be crushed or decapsulated were considered unable to be administered using a tube. Powdered drugs were tested in the same way. For cytotoxic drugs including teratogenic and toxic drugs, caution was taken not to touch the drug directly, and cracking and decapsulation were not performed. Otherwise, they were tested in the same manner as that for non-cytotoxic drugs. A total of 3686 tablets and 432 capsules used frequently in Japan were tested. A comprehensive examination of drugs provided by various companies and leftover drugs was performed.

### Tube Passage Test

**Fig. 1:**
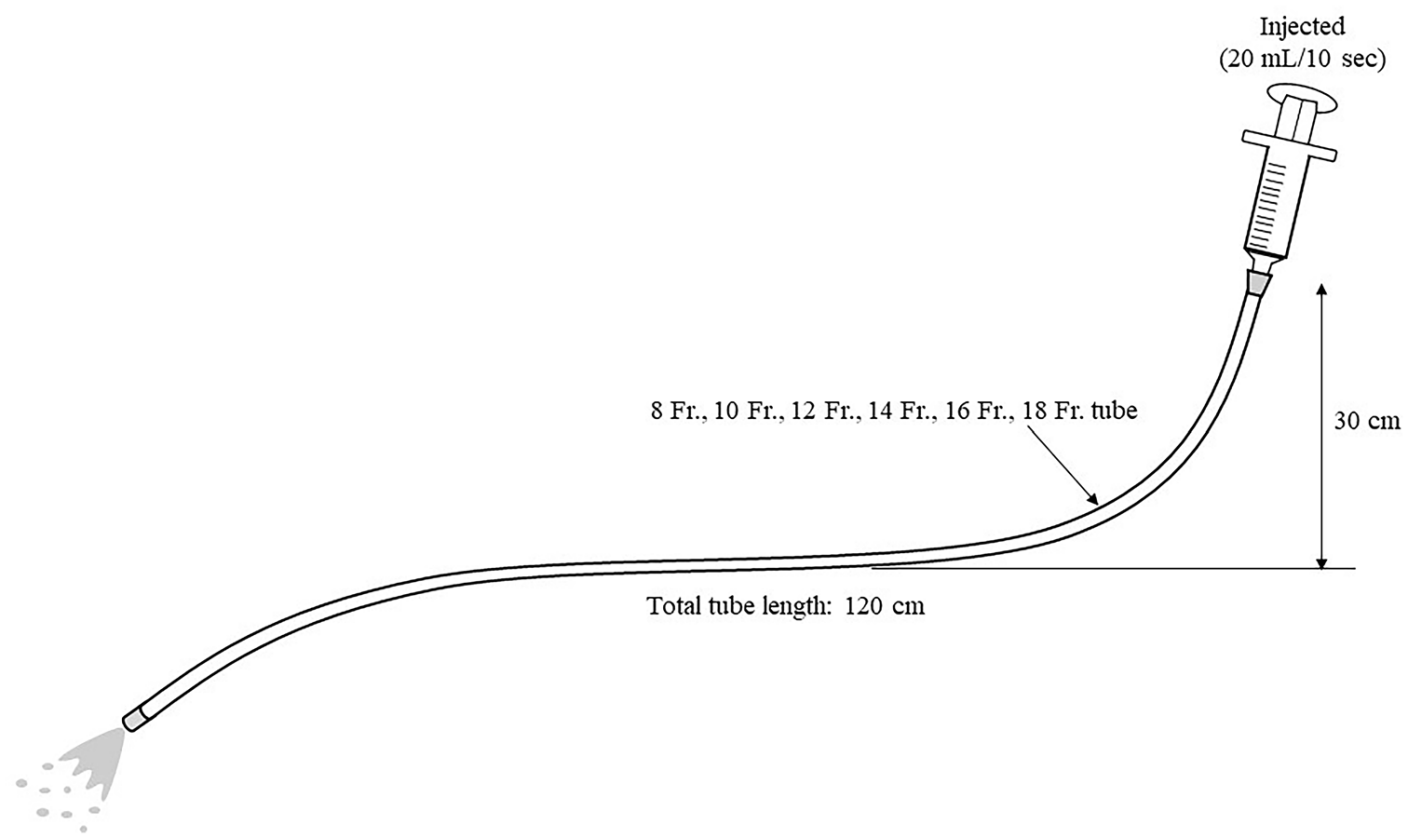
Tube passage test. Placing the tube in a bedridden patient with the distal two-thirds lying flat and the proximal side at a height of 30 cm

The 20 mL drug suspension liquid was expelled from the syringe into the nutrition tube manually over 10 s. The tube was placed as it would have been done for a bedridden patient (Fig. 1). The formula was passed through tubes of different sizes (8 Fr., 10 Fr., 12 Fr., 14 Fr., 16 Fr., and 18 Fr.) that are often used in clinical settings in Japan. Tube passage tests were performed in a fixed order, starting with the 8 Fr. tube. After passing the formula, using the same syringe, 20 mL of water was measured and passed. The tubes were inspected visually for residual drug, and if no noticeable residue or pieces were identified, it was judged to have passed the tube passage test. These two steps were performed by two pharmacists. The percentage of drug that passed the tube passage test was determined.

This study was approved by the Ethics Committee of Hamamatsu City Rehabilitation Hospital (#20-15).

## Results

Three thousand six hundred and eighty-six tablets and 432 capsules underwent the suspension test and tube passage test. A part of the results is shown in Table 1.

**Table 1:** Example of the results of a simple suspension method

Proprietary name	Contents (coating)	Formula characteristics	Minimum Passage Diameter	55℃ 5 min	55℃ 10 min
Itorol	20 mg (naked)		8 Fr.	○	○
IPD	100 mg (Cap)	White powder	8 Fr.	○	
Aspara-CA	200 mg (naked)		8 Fr.	×	×
Azeptin	2 mg (sugar)		14 Fr.		○

○, both disintegrated within 10 min and passed the tube passage test

×, either not disintegrated within 10 min or passed the tube passage test

Of the 3686 tablets, 3377 tablets (91.6%) disintegrated within 10 min and passed the tube passage test (Table 2). Three hundred and nine tablets (8.4%) were not administrable. In contrast, with the crushing method, 2117 tablets (57.4%) of the 3686 tablets could be crushed, according to the interview forms of each tablet.

**Table 2:** Results of the tube passage test

	Tablet (*n* = 3686)	Capsule (*n* = 432)
SSM		
Administrable	3377 (91.6%)	359 (83.1%)
Not administrable	309 (8.4%)	73 (16.9%)
Crush		
Administrable	2117 (57.4%)	272 (63.0%)
Not administrable	1569 (42.6%)	160 (37.0%)

*SSM* simple suspension method

Of the 432 capsules, 359 capsules (83.1%) disintegrated within 10 min and passed the tube passage test. Seventy-three (16.9%) capsules did not pass the tube. On the other hand, with the decapsulated method, 272 capsules (63.0%) of the 432 capsules could be decapsulated, according to the interview forms of each capsule.

Some tablets and capsules could be administered with a wider tube. The full list of the drugs studied can be seen in the tube administration of drugs handbook [15].

## Discussion

This is, to the best of our knowledge, the first study published in English to report that a large number of drugs are suitable for administration using SSM. SSM can solve numerous issues associated with crushing tablets. Furthermore, the number of drugs indicated for SSM is greater than that indicated for the conventional crushing method. A strength of this study is that the effectiveness of SSM was examined with a large number of drugs. In Japan, SSM is widely used in patients diagnosed with dysphagia. Our hypothesis was verified in this study.

The most important finding in our study is that SSM decreases the risk of clogging nutrition tubes. Wider tubes can be used for drug administration; however, thinner tubes are recommended whenever possible, as wider tubes can obstruct swallowing and increase the patient’s discomfort [16]. Tube placement has a negative effect on swallowing function, particularly in the pharyngeal phase of swallowing, and it increases the risk of pharyngeal residue and aspiration [17, 18]. Furthermore, nasogastric tube syndrome is more likely to occur with wide tubes [19]. Thus, it is important to use a thinner tube for patients diagnosed with dysphagia. SSM is useful for such patients who need a thin nasogastric tube such as the 8 Fr. tube.

In SSM, changes in the physical characteristics of the drugs could be reduced compared to those with the conventional crushing method. Crushed tablets can be vulnerable to changes in light, temperature, and humidity, and their efficacy may be affected physiochemically. Changes upon mixing may occur when multiple crushed tablets are packaged together. In SSM, tablets and capsules are maintained in their original form until immediately prior to administration. Thus, these effects are eliminated. However, little is known about the changes that occur during disintegration, although the same issue arises when suspending crushed drugs in water. Further studies are needed in this regard.

Of note, SSM reduces the loss of the drug when physicians need to change or cancel prescribed medicines. Tablets and capsules are maintained in their original form, so it is easy to accommodate the changes in prescribed medicines. Furthermore, the economic burden can also be decreased because drugs need not be discarded. When drugs are crushed, the person preparing the drug may touch or inhale the drug and be prone to drug-related health hazards. This can be avoided with SSM. Compared to crushing the drugs, the preparation is easier, the required time is shorter, and the workload of healthcare professionals and caregivers is reduced. Drugs that are not stable at 55 °C are not suitable for this method of administration. Interview forms of each drug should be taken into account.

SSM is also effective for dysphagic patients who cannot take tablets by mouth. Thickening the simple suspension of tablets allows these patients to take the disintegrated drugs. Thus, SSM might be safe and clinically effective as a route of administration of drugs for patients diagnosed with dysphagia in acute care hospitals including intensive care units, chronic care hospitals, and home care facilities [15].

This study had some limitations. First, enteric-coated tablets and sustained-release tablets may be administered if they are provided as multiple units and the granules in the tablet can pass through the tube. On the contrary, single-unit enteric-coated or sustained-release tablets cannot be cracked; therefore, they were assigned to the ‘not administrable’ group without further testing in this study. Moreover, these drugs cannot be administered by the conventional crushing method. Second, the stability of the drugs in water at 55 °C remains uncertain. However, there have been no reports of adverse events resulting from the administration of SSM in clinical settings. Third, this study was limited to Japan. The efficacy of the administration of drugs via SSM needs to be verified in more countries.

In conclusion, SSM reduces the risk of tube blockage and drug loss with a large number of drugs compared with the conventional crushing method. However, further studies are required to evaluate its utility compared to that of the conventional methods that are currently in use for treating patients diagnosed with dysphagia in actual clinical settings.
